# The Interplay between Nutrition, Innate Immunity, and the Commensal Microbiota in Adaptive Intestinal Morphogenesis

**DOI:** 10.3390/nu13072198

**Published:** 2021-06-26

**Authors:** Franziska Bayer, Olga Dremova, My Phung Khuu, Könül Mammadova, Giulia Pontarollo, Klytaimnistra Kiouptsi, Natalia Soshnikova, Helen Louise May-Simera, Kristina Endres, Christoph Reinhardt

**Affiliations:** 1 Center for Thrombosis and Hemostasis (CTH), University Medical Center of the Johannes Gutenberg-University Mainz, Langenbeckstrasse 1, 55131 Mainz, Germany; franziska.bayer@uni-mainz.de (F.B.); odremova@students.uni-mainz.de (O.D.); mkhuu@students.uni-mainz.de (M.P.K.); kmammado@students.uni-mainz.de (K.M.); giulia.pontarollo@unimedizin-mainz.de (G.P.); Klytaimnistra.Kiouptsi@unimedizin-mainz.de (K.K.); 2 Institute of Molecular Medicine, University Medical Center of the Johannes Gutenberg-University Mainz, Langenbeckstrasse 1, 55131 Mainz, Germany; soshniko@uni-mainz.de; 3 Faculty of Biology, Institute of Molecular Physiology, Johannes Gutenberg-University Mainz, 55128 Mainz, Germany; may-simera@uni-mainz.de; 4 Department of Psychiatry and Psychotherapy, University Medical Center of the Johannes Gutenberg-University Mainz, 55131 Mainz, Germany; kristina.endres@unimedizin-mainz.de; 5 German Center for Cardiovascular Research (DZHK), University Medical Center of the Johannes Gutenberg-University, Mainz Parter Site Rhine-Main, Langenbeckstrasse 1, 55131 Mainz, Germany

**Keywords:** intestine, development, morphology, microbiota, immunometabolism, vascularization, endothelium, epithelial cells, enteric nervous system, nutrition, high-fat diet

## Abstract

The gastrointestinal tract is a functionally and anatomically segmented organ that is colonized by microbial communities from birth. While the genetics of mouse gut development is increasingly understood, how nutritional factors and the commensal gut microbiota act in concert to shape tissue organization and morphology of this rapidly renewing organ remains enigmatic. Here, we provide an overview of embryonic mouse gut development, with a focus on the intestinal vasculature and the enteric nervous system. We review how nutrition and the gut microbiota affect the adaptation of cellular and morphologic properties of the intestine, and how these processes are interconnected with innate immunity. Furthermore, we discuss how nutritional and microbial factors impact the renewal and differentiation of the epithelial lineage, influence the adaptation of capillary networks organized in villus structures, and shape the enteric nervous system and the intestinal smooth muscle layers. Intriguingly, the anatomy of the gut shows remarkable flexibility to nutritional and microbial challenges in the adult organism.

## 1. Introduction

During embryonic development, the gastrointestinal tract is patterned into functionally distinct segments that constantly undergo adaptive remodelling processes [1,2]. For the entire life span of an organism, the gastrointestinal tract withstands constant exposure to manifold environmental stressors [3]. Upon birth, this organ governs the uptake, transport, and digestion of nutrients, but it also has a pivotal role in metabolic regulation and the efficient excretion of catabolites and fibers [4].

*In utero*, the mammalian fetus is generally believed to develop in a sterile environment. Remarkably, since bacteria were recently identified in the amniotic fluid, the placenta, and meconium, it is currently under debate whether microbial colonization of the fetus already initiates prior to gestation [5,6,7]. At birth, the gastrointestinal tract (as well as other body surfaces) is colonized by microorganisms from the environment [8,9]. This results in the formation of a densely populated microbial ecosystem, termed gut microbiota, that exist in a mutualistic relationship with its host, influencing the development and tissue homeostasis of its habitat. The mammalian gut microbiota is dominated by anaerobic bacteria belonging to the Bacteroides and Firmicutes phyla [10]. In addition to the intestinal microbiome, the oral microbiome represents a highly diverse ecosystem that likewise affects mucosal remodeling processes [11,12].

Directly after birth, gut bacteria promote intestinal immune maturation, for example through the regulation of epithelial synthesis of retinoic acid and RORγt^+^ type 3 innate lymphoid cells, that are required for the formation of cryptopatches and intestinal lymphoid follicles to fight the invasion of pathogens [13]. Furthermore, there is increasing evidence linking changes in the maternal gut microbiota to consequences of pregnancy and newborn health [14,15]. Disturbances affecting the composition of this microbial ecosystem (dysbiosis), often occurring in concert with pathobiont colonization or antibiotic therapy, can be causative of inflammatory immune recactions (e.g., in inflammatory bowel disease) [16]. However, dysbiosis can also be caused by nutrition, such as a Western-type diet, thus favoring the development of autoimmune diseases [17,18]. Hence, in early life, the microbiota affects immune maturation by various metabolites.

Due to its adaptive nature [19], the gastrointestinal tract is robust enough to warrant the co-existence with a highly diverse and densely populated microbial ecosystem, the commensal gut microbiota [10]. Microbiota–host interactions in the gut mucosa impact immune functions while ensuring peripheral tolerance [20,21]. For instance, polyamines, which are L-arginine metabolites derived from nutritional sources and gut commensals, promote the development of regulatory T cells in the intestine [22,23]. Intriguingly, microbial metabolites integrate into and interfere with host metabolism [24,25]. Besides being important determinants of immune functions, these microbial metabolites can influence the physiology of remote organs [26,27,28].

Interestingly, vast adaptation of various morphological features of the gut can be observed following colonization with gut commensals. Major colonization-dependent changes in gut morphology are enhanced epithelial renewal via stimulation of innate immune pathways [29,30], postnatal development of capillary networks and lacteals in the villus structures of the small intestine [31,32,33], and shaping of the enteric nervous system (ENS) [34].

Compared to the roles of microbiota and diet in intestinal immune homeostasis [35], the involvement of non-immune cells in the context of intestinal tissue adaptation remains poorly resolved. Hence, our review puts the focus on the current insights on the morphometric adaptation of the intestine, gained by mouse models. Representing genetically modifyable mammals that can be kept in germ-free housing conditions [36], mice are the most prominent in vivo models to investigate mammalian gut development and the impact of human-relevant diets. We provide a comprehensive overview on current insights of how diet and gut commensals affect the cellular plasticity of the intestine, including the remodelling of villus capillaries and the enteric nervous system, resulting in adaptation of gut morphology.

## 2. Development of Intestinal Tissues

### 2.1. General Aspects and Early Gut Development

In mice, having a 19-day gestational period, the heart and the alimentary tract of the embryo are initially housed within a common coelom, which is then subdivided into pericardial and peritoneal cavities by the septum transversum at embryonic day 8.5 [37]. The alimentary canal is encased in the peritoneal coelom. During gastrulation, starting at embryonic day 6.25 and completed by embryonic day 9.5, the endoderm and the splanchnic mesoderm give rise to the gastrointestinal tract [38]. At embryonic day 8.5, the gut consists of two epithelial layers, the endoderm and the mesoderm. Tube closure is complete at embryonic day 9 [38]. At embryonic day 9.5–10.5, the cells of the gut mesoderm proliferate and divide into several layers. A third germ layer, the ectoderm, innervates the gastrointestinal tract [39]. During this process, the single-layered pluripotent epiblast, a cup-shaped epithelial layer emerging at embryonic day 3.5 *post coitum* in the mouse blastocyst, undergoes epithelial to mesenchymal transition, forming definitive endoderm, mesoderm, and ectoderm [40]. The definitive endoderm gives rise to the epithelium of the gastrointestinal tract, thymus, thyroid, and respiratory tract. The mesoderm gives rise to the cardiovascular system, muscles, blood, and bone. The ectoderm develops into the skin epithelium and the central nervous system.

Interestingly, the gut develops into distinct functional domains, both in the anterior-posterior direction and in the cross-sectional axis. From proximal to distal, the gastrointestinal tract can be subdivided into three different regions: (1) the foregut, consisting of the esophagus, stomach, proximal duodenum, thymus, thyroid, airways, pancreas, and the liver; (2) the midgut, comprising the distal duodenum, jejunum, ileum, cecum, and the ascending and proximal transverse colon; (3) the hindgut, composed of the transverse colon, descending colon, sigmoid, and anorectum. Various morphogen gradients control the interaction between the germ layers during gut development [1]. Conceptually, these morphogenetic signals control three fundamental processes: (1) regionalization of the gut tube, meaning that distinct regions with different functions are formed along the anterior-posterior axis [41,42,43]; (2) radial patterning of the tube, achieving proper placement of the epithelium, connective tissues, muscle layers, nerve plexuses, vascular and lymphatic vessels, and glands; (3) continuous and persistent self-renewal of the gastrointestinal epithelium from stem cells is ensured [44].

### 2.2. Development of the Intestinal Epithelium and Villus Formation

On embryonic day 9.5 in mouse development, the gut tube becomes a simple, pseudostratified epithelium with most cells touching both, apical and basal surfaces [45]. From embryonic day 9.5–13.5, the epithelium and the mesenchyme rapidly proliferate. During this period, the gut tube elongates and the gut circumference and lumen increase [46,47]. This process involves the wingless/Int-1 (Wnt) and the hedgehog (Hh) signaling pathways [48,49]. Between embryonic day 12.5 and embryonic day 14, the tightly packed pseudostratified epithelium develops into a stratified epithelium with apical cells (luminal) connected by junctional complexes [50]. At embryonic day 14, the stratified epithelium of the midgut and the hindgut endoderm reorganize into a simple columnar epithelium, covering the luminal surface of the intestine [51].

In the small intestine, the epithelium is organized in finger-like protrusions, so-called villus structures, and invaginations, the crypts of Lieberkühn, in order to maximize the surface for nutrient absorption. The large intestine, on the other hand, lacks these villi, while retaining the crypts. The formation of finger-like epithelial protrusions into the lumen of the small intestine is observed at embryonic day 14.5, instructed by epithelial-derived Hh and platelet-derived growth factor (Pdgf) signals acting on the gut mesenchyme [52,53,54]. At embryonic day 15, the first villus structures emerge [51]. In contrast to avian villus formation, the zigzag stage, a characteristic of the progressive bending that is driven by sequential differentiation of smooth muscle layers, is entirely missing in mammals and villus structures emerge directly from a flat epithelium. In fact, mesenchymal invasion is the first sign of villus emergence [55,56]. Villus outgrowth is initiated from tight clusters of mesenchymal cells that form *de novo*, acting as signaling centers [53]. Clustered mesenchymal cells show robust expression of the Hh targets gli-family zinc finger 1 (Gli-1) and patched 1 (Ptch-1), induced by paracrine Hh signals. Indeed, inhibition of the Hh pathway at embryonic day 13.5 prevents the formation of clusters and villus structures [52]. The central role of the Hh pathway for villus formation is supported by several independent mouse studies [49,57]. In addition, this process is believed to depend on paracrine signaling between platelet-derived growth factor expressed by the pseudostratified epithelium and platelet-derived growth factor receptor-α expressed by subepithelial mesenchymal cells, promoting the proliferation of the underlying mesenchymal cells. Bone morphogenetic protein (BMP) signals derived from clustered mesenchymal cells inhibit the cell cycle of the cluster and its associated epithelium [53,58]. After cluster formation, the epithelial cells situated above the clusters stop proliferating and begin to differentiate. In this way, proliferation is restricted to the intervillus epithelium, which will host the intestinal crypts. As the epithelium converts to a columnar shape at mesenchymal clusters, the epithelial cells above these clusters rapidly shorten and widen [59]. Interestingly, from embryonic day 16, the fetal mouse colon develops villus-like structures before adopting the deeper crypts and flat intercrypt table of mature colonic epithelium. Over a 36-hour time period, villus structures first arise in the duodenum and then emerge in the ileum [52]. Intervillus regions develop into flask-like crypts only after birth [51].

In mice and humans, villus development and muscle layer maturation are not concomitant [58]. The inner circular muscle layer forms at embryonic day 12, the outer longitudinal muscle layer at embryonic day 15, and the muscularis mucosa muscle layers at embryonic day 18, later than the initiation of villus formation. BMPs are primarily expressed in the mesenchyme and regulate spacing and patterning of mesenchymal clusters, depending on BMP receptor 1-α expression by these cell clusters [58]. Starting at embryonic day 16.5, cytodifferentiation of the villus epithelium occurs, giving rise to a multitude of epithelial cell types, each having a distinct function (secretory and absorptive lineage) [2]. However, the participation of vascular- and neuronal-epithelial crosstalk in intestinal morphogenesis remains poorly explored [59,60]. At birth (embryonic day (E) 18.5 in the C57BL/6J mouse strain), the cellular and structural features of the intestine are developed but anatomical and functional maturation and adaption take place dependent on various environmental conditions (i.e. nutrition, presence of microbiota).

### 2.3. Intestinal Vasculogenesis

**Figure 1:**
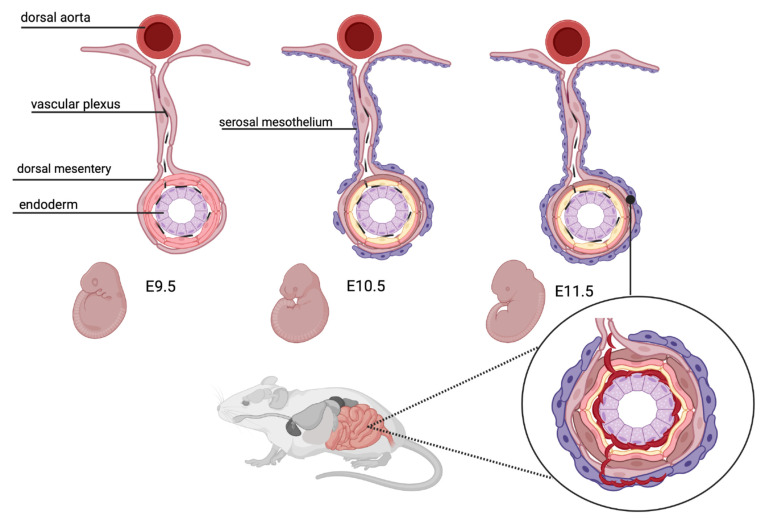
Vascular development of the mouse intestine. Between embryonic day (E) 9.5 and E11.5, endothelial cells from the serosal mesothelium innervate the intestine in a proximal to distal manner. Created with *BioRender* [63].

In the mature small intestine, the villus vasculature consists of a centrally situated lymphatic vessel, the lacteal, which is surrounded by intricate capillary networks that are in close proximity to the villus epithelium. In the developing mouse intestine, the vasculogenic cells originate from the serosal mesothelium [61]. The developing gut is initially devoid of mesothelium but serosal mesothelial cells migrate into the gut. At embryonic day 9.5, platelet endothelial cell adhesion molecule-1 (PECAM1)-positive endothelial tubes appear in the proximal half of the intestine with sprouting endothelial cells in the distal intestine [62]. At embryonic day 10.5, mesothelial cells are detected at the proximal base of the dorsal mesentery. From embryonic day 10.5 onwards, serosal mesothelial cells appear in a proximal to distal manner, enclosing the embryonic gut by embryonic day 11.5 [61] (Figure 1; created with *BioRender* [63]). Beginning at embryonic day 13.5, tube-forming endothelial cells extend from the vascular plexus to the surface of the gut. Although these endothelial cells penetrate the mesodermal wall, they are devoid of smooth muscle cells. This capillary network undergoes extensive angiogenic remodeling and reorganizes to form the characteristic, hierarchically branched enteric vasculature by embryonic day 15.5 [62]. At this stage, arteries and veins can be distinguished by morphology and characteristic markers (ephrin B2 for arteries; ephrin B4 for veins). External *vasa recta* are readily identified along the length of the small intestine, branching into the submucosal layer [62]. Smooth muscle actin-positive cells appear at embryonic day 12.5 in the developing mesentery. While they surround intestinal arteries until embryonic day 15.5, there is no coverage of veins or microvasculature in the intestinal submucosa [62].

From embryonic day 16.0 and later, endothelial cells in part undergo endothelial-to-mesenchymal transition, differentiating into the smooth muscle cells of the mesentery and the intestine [61]. By embryonic day 18.5, mesothelial cells are developed in the entire gut tube and the mesentery. Of note, the serosal mesothelium not only gives rise to the endothelium, but contributes to the vast majority of vascular smooth muscle cells and other non-vascular cells in the intestine.

At a later stage, the lymphatic vasculature develops from the mesentery, with its endothelial cells first appearing adjacent to the superior mesenteric artery, reaching the duodenum at embryonic day 13.5 and the ileum at embryonic day 15.5 [62]. At embryonic day 16.5, lymphatic vessels surround all major vessels in the submucosa of the duodenum, and the remodeled vessels may be functionally linked to lymphatic development. Interestingly, it has been demonstrated that the separation of the intestinal mucosal lymphatic and blood microvasculature is a process whose regulation continues postnatally, involving fasting-induced adipose factor (FIAF) [64].

### 2.4. Development of the Enteric Nervous System

A unique feature of the gastrointestinal system is the presence of an intrinsic nervous system, independent from the central nervous system. In the adult intestine, the enteric nervous system (ENS) harbors two major ganglionized networks embedded in the gut wall, i.e., the myenteric plexus (Auerbach plexus) responsible for motility and peristalsis, and the submucous plexus (Meissner plexus) involved in the regulation of gland secretion and interconnected with the gastrointestinal immune system [65]. While the myenteric plexus and the submucous plexus are quite distant to the gut lumen, the enteric glia within the submucous layer are in contact with the gut epithelial lining [66].

The ENS represents the largest and most complex part of the peripheral nervous system [67]. In embryonic vertebrate development, somites appear temporarily as two paired strands of mesoderm situated right and left of the *chorda dorsalis* and the neural tube. They originate from the paraxial budding of the mesoderm. The neurons and the glial cells of the ENS originate from the vagal neural crest (somites 1–7) [68,69]. At embryonic day 9.5, neuronal crest cells migrate into the stomach, propagate, and differentiate into the enteric plexus [68,70], colonizing the entire gut by embryonic day 14. An additional origin of the ENS are cells derived from sacral neural crest cells at the level of the 28th somite reaching the hindgut via the pelvic plexus [71]. Of note, neuronal crest-derived cells arrive in the proximal intestine directly after the formation of the primary capillary plexus, but before angiogenic remodelling takes place [62]. The migration of neural-derived crest cells may involve soluble signals from capillary endothelial cells [72].

During the colonization period, massive proliferation of neural crest cells occurs, with differentiation into either glial cells or various cells of the neuronal lineage. ENS development is regulated by the transcription factor achaete-scute complex homolog 1 (ASCL1; formerly named mouse achaete-scute homolog 1 (MASH1)) in the esophagus, glial-derived neurotrophic factor (GDNF) in the gastrointestinal tract caudal to the stomach, and endothelin 3 and endothelin receptor B in the hindgut [73]. According to the expressed neurotransmitters, ENS neurons can be subdivided into cholinergic, nitrergic, calretinin-expressing, and neuropeptide-expressing neurons, as well as catecholaminergic and inhibitory gamma-amino-butyric acid (GABA) neurons [74]. The first enteric neuronal precursors differentiate between embryonic day 8 and 10.5 into cholinergic cells. Later on, other neuronal subtypes differentiate, for instance, the serotonergic cells at embryonic day 11.5, the tyrosine hydroxylase-positive and nitric oxide synthase 1-positive neurons at embryonic day 15.5, and the calretinin-positive neurons at birth [75,76,77].

One interesting feature of the ENS is that this system undergoes continuous renewal even in adult life. The neural cells expressing the neuroepithelial stem cell marker nestin persist in the adult intestine, giving rise to enteric neurons and glial cells, e.g., in response to injury of the adult ENS [78,79]. Furthermore, there is increasing evidence for interrelation between neurogenesis and vasculogenesis during gut development [80,81].

## 3. Intestinal Epithelial Homeostasis and Renewal

The intestinal mucosa is composed of two components: a polarized, selective-permeable monolayer of epithelial cells lying above a basement membrane and an underlying connective tissue, the so-called lamina propria. In addition to the epithelial lining, endothelial cells and immune cells also contribute to the gut barrier function [82,83,84]. The intestinal epithelial cell types are distinguished into two lineages, defining the main functions of the intestine: cells of the absorptive and the secretory lineage. The simple absorptive enterocytes compose 80% of the epithelial lineage. The secretory cells are mucin-secreting goblet cells, hormone-producing enteroendocrine cells, and Paneth cells, releasing anti-microbial peptides [85]. At the villus tip, apoptotic, damaged, infected or otherwise compromised cells are shed into the gut lumen, a process called anoikis [86,87]. Hence, both in humans and mice, the intestinal epithelium is completely renewed every three to four days [88].

### 3.1. Epithelial Lineage Commitment in the Self-Renewing Crypt-Villus Unit

The epithelium of the small intestine is organized into a myriad of crypt-villus units. Six independent intestinal stem cells (ISC) reside with Paneth cells in the crypt, whereas all other epithelial cell types are localized in the villus epithelium [85]. According to one model, the ISC are located at the +4 position on top of three Paneth cells at the crypt bottom [19,89]. A second model, defining ISC as crypt-base columnar (CBC) cells, states that they are squeezed in between the Paneth cells at the bottom of the crypt [90]. Leucine-rich-repeat-containing G-protein-coupled receptor-5 (Lgr-5) was identified as a highly specific ISC marker that is only expressed in CBC cells [91]. Mouse intestinal Lgr5+ stem cells divide symmetrically into transient-amplifying (TA) cells [92]. Of note, intestinal villus structures receive epithelial cells from multiple crypts throughout life (they are polyclonal). The proliferative activity of the intestinal epithelium is driven by the Wnt-pathway via the TA cells in the crypts. As mentioned before, patterning of the crypt-villus axis and epithelial hyperproliferation is controlled via the Hh pathway [54]. Furthermore, the number and the proper development of villus structures are influenced by platelet-derived growth factor-α (PDGF-α) signaling and the BMP pathway [53].

During lineage commitment, TA cells, an undifferentiated population in transition between stem cells and differentiated cells, migrate upwards along the crypt-villus axis and terminally differentiate into one of the four principal epithelial cell lineages. The secretory lineage consists of (i) mucin-secreting goblet cells, (ii) enteroendocrine cells, releasing hormones, and (iii) Paneth cells, which produce antimicrobial peptides and are the only cell type migrating to the bottom of the crypts [19]. The fourth cell type is the absorptive enterocyte. In addition to the Wnt-pathway, the Notch-pathway is critical in maintaining the crypt compartment in its undifferentiated state. If Notch-signaling is blocked, this results in the rapid and complete conversion of all epithelial cells into goblet cells [93,94]. Additional cell types, such as microfold cells (M cells), Tuft cells, and Brush cells were described (Table 1).

**Figure 2:**
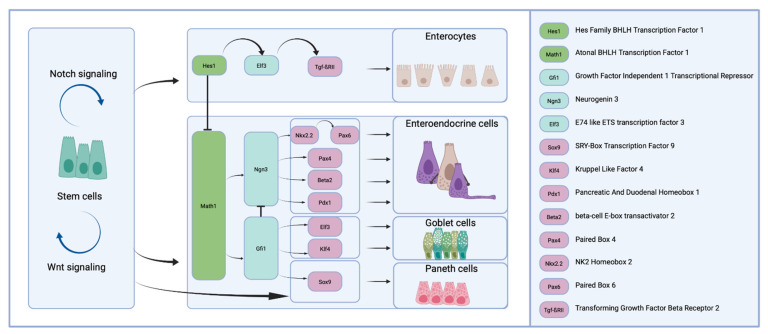
Differentiation factors involved in epithelial lineage commitment. Created with *BioRender* [63].

Importantly, a strict genetic hierarchy in cell lineage commitment is found in the intestine (Figure 2), counterposing two major genes: hairy/enhancer of split-1 (Hes-1) and Atoh1 (formerly named Math1). Indeed, the direct Notch-target gene Hes-1 represses transcription of the transcription factor Atoh1 [95]. In Hes-1 positive cells, the expression of E74-like ETS transcription factor (Elf3) and Tgf-βRII drives enterocyte development (absorptive lineage). Atoh1 activity promotes differentiation into goblet, enteroendocrine, and Paneth cells (the secretory lineage). Krüppel-like factor 4 (KLF4) and Elf3 promote differentiation into goblet cells, whereas neurogenin 3 (Ngn3) is required for the development of the enteroendocrine lineage [19]. Growth factor independent 1 transcriptional repressor (Gfi1) and SRY-box transcription factor 9 (Sox9) are required for the differentiation into Paneth cells, which serve innate immune functions, such as the secretion of lysozyme and the release of defensins [96,97]. Their maturation requires active Wnt signaling. Delta-like ligand expression by Paneth cells (Dll1^+^, Dll4^+^) triggers Notch1 and Notch2 in stem cells, which results in the repression of the Atoh1 transcription factor, thus the Lgr5^+^ stem cells are prevented from differentiation into the secretory lineage [98,99].

Goblet cells are mucus and trefoil protein secreting cells required for movement and expulsion of gut contents [100].

**Table 1:** Location and functions of intestinal epithelial cell types.

Cell Type (Ref.)	Localization	Histomorphology	Key Functions
Enterocyte [101]	Small intestine, large intestine Along villus	Columnar polarized cell with basal oval nucleus Apical brush border (Microvilli)	Absorption of nutrients, water Gut barrier Secretion of antimicrobial peptides Interaction with innate immune system
Goblet cell [100]	Small intestine, Colon Along villus	Columnar cell, apical part enlarged Small triangular nucleus basal Cytoplasm with secretory granules (mucins)	Secretion of mucins and other glycoproteins Nonspecific endocytosis of antigens Interaction with innate immune system
Enteroendocrine cell [102]	Small intestine, Colon Along villus	Shape varies with cell subtype, based on secreted hormones Long basal processes to interface with neurons or adjacent intestinal epithelial cells	Secrete hormones (e.g., serotonin) Detect gut microbes and microbial metabolites Interaction with innate immune cells
Tuft cell [103,104]	Small intestine, Colon Along villus	Cylindrical cell body, narrows on apical and basal end Highly organized brush border Lateral membrane projections to adjacent enterocytes	Innate immune responses to helminth infection Contribute to epithelial regeneration
Microfold cell [105]	Small intestine Above gut associated lymphatic tissue (GALT)	Columnar cell No apical brush border Basal interaction with lymphocytes/dendritic cells	Capture luminal antigen and present it to immune cells Inflammation can induce development of microfold cells
Paneth cell [96]	Small intestine Crypts	Large eosinophilic secretory granules	Secrete antimicrobial peptides Regulating stem cell niche

**Figure 3:**
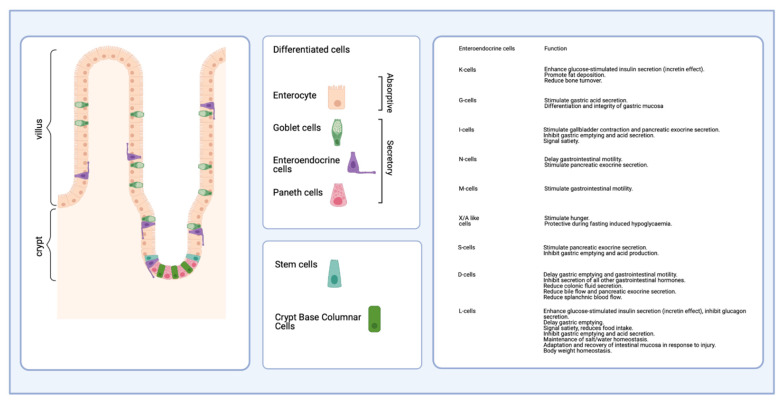
Functional overview on the enteroendocrine cell types of the secretory lineage. Created with *BioRender* [63].

The proportion of goblet cells increases from the duodenum (4%) to the descending colon (16%) [106]. Furthermore, Tuft cells serving immune functions and M cells, the cells overlying the lymphoid follicles of maturing Peyer’s patches are present [103,104]. In total, the enteroendocrine cells consist of 15 different subtypes representing approximately 1% of the epithelial cell count, which are classified according to the produced hormones [105] (Figure 3). They are distributed along the columnar gut epithelium, regulate intestinal function and interfere with energy metabolism by the secretion of peptide hormones [107]. The enterocytes are epithelial cells of the absorptive lineage. They are highly polarized cells with an apical brush border (microvilli), ensuring the selective absorption and transport of nutrients through the epithelial lining [101]. In total, they make up 80% of all intestinal epithelial cells. As such, a multitude of physiological functions is fulfilled by an adjusted differentiation of the epithelial lineage.

### 3.2. The Functional Role of the Intestinal Mucus Layer

Goblet cells secrete mucins and peptides of the trefoil factor family, which are important factors inducing mucosal healing [108]. Of note, the mucus layer is composed of two distinct layers, an inner layer, which is adherent to the epithelial cells, and a more diluted outer layer. Its main function is the smooth transport of luminal content but also the separation of gut bacteria from the epithelial lining [109]. However, a number of bacteria can invade the mucus layer and come into contact with epithelial cells [110,111,112]. The inner layer is rich in antimicrobial peptides and mucins, whereas the outer layer is more diluted, so gut bacteria may reside in it. Thickness and composition of the mucus layer vary along the gut: in the small intestine, only the outer layer is present, whereas, in the colon and caecum, the sites with the highest bacterial colonization density, both layers are fully developed [113,114,115]. Mice deficient in Mucin 2 (Muc2), a gene encoding for the major mucus protein, develop spontaneous intestinal inflammation, probably as a result of the missing mucus layer and the prolonged contact between gut bacteria and intestinal epithelium [116].

## 4. Nutrition, Microbiota, and Innate Immune Signaling Adapts Gut Morphology and Cell Homeostasis

In addition to the genetic determinants detailed above, environmental factors also contribute to gut development and renewal. In the next sections, we will discuss about how nutrition, the gut microbiota, and the innate immune signaling impact these processes.

### 4.1. Effects on Epithelial Lineage Commitment and Renewal

Nutrition impacts gut homeostasis. On one side, intestinal stem cells are directly influenced by dietary factors as well as by-products of bacterial fermentation of dietary fibers, such as short-chain fatty acids (SCFAs). On the other side, the impact of diet on the composition of the gut microbiome, with changing levels of bacterial metabolites, also affects intestinal stem cells.

#### 4.1.1. Impact of Nutrition

The impact of nutrition is predominantly addressed by studying specific mouse models with feeding regimens recapitulating human diets. In the early 1990s, Newmark and coworkers found that colonic hyperproliferation and hyperplasia in rats and mice is induced by a Western-style diet, which was designed to mimic four human risk factors for colon cancer (high dietary fat, low calcium levels, reduced Vitamin D3 intake, and increased phosphate levels). After twelve weeks of exposure to this dietary stressor, longer colonic crypts (hyperplasia) and higher numbers of 3H-thymidine labeled epithelial cells (hyperproliferation) were found in these rodents [117,118]. Recently, using the same diets, Li and coworkers established that Lgr5+ cells were reduced in function and number in these animals [119]. Transcription of Lgr5+ cells was altered, particularly affecting the DNA mismatch repair pathway. To compensate for the reduction of Lgr5+ cells (BMI1 polycomb ring finger proto-oncogene), Bmi1+ cells were mobilized to take over their stem cell function. The same researchers also found that the Lgr5+ cells are highly sensitive to altered calcium and vitamin D3 levels. These findings are in line with previously published data, showing that lower vitamin D3 and calcium levels inhibit cell maturation, Wnt signaling, and reduce progenies of Lgr5+ cells [120,121,122].

In more recent studies, variations of the HFD were tested in mice. Cheng and coworkers fed mice an HFD low in carbohydrates (ketogenic), leading to an upregulation of the 3-hydroxy-3-methylglutaryl-CoA synthase 2 (HMGCS2) protein expression [123]. HMGCS2 is expressed in small intestinal Lgr5+ stem cells and produces ketone bodies, providing energy during fasting [124]. β-hydroxybutyrate, a ketone body produced by HMGCS2, facilitates Notch signaling as a signaling-active metabolite, thereby increasing the intestinal stem cell number andfunction, and promoting intestinal injury repair after radiation damage [124]. Ketogenic diets (diets with low carbohydrate content, 5–10 % of total caloric intake) also impact the composition of the gut microbiota, decreasing total diversity and altering the abundance of several bacterial species [125,126].

In addition, single molecules may have a strong impact on gut morphology. The n-3 polyunsaturated fatty acid (PUFA) α-linolenic acid (ALA) has anti-inflammatory, anti-carcinogenic, and anti-obesogenic properties [127,128,129]. It is also reported that α-linolenic acid has a direct impact on intestinal stem cell differentiation, as PUFA-rich diet-fed rats presented decreased amounts of goblet cells in the colon [130]. Research by Todorov and coworkers describes increased mucosal thickness, villus length in the small intestine of ALA-rich diet- and HFD diet-fed mice [130]. While the numbers of enterocytes were increased, mice fed with either ALA or HFD diet had decreased numbers of cells of the secretory lineage (Paneth and goblet cells), in agreement with previous studies, as well as decreased numbers of proliferating Ki67-positive cells [131].

Glutamine, an amino acid mainly produced in muscle cells, adipose tissue, lung, and brain, also serves as an energy source in enterocytes [132,133]. Dietary supplementation of glutamine promotes proliferation in porcine intestinal epithelial cells, stimulating crypt cells to enter the S-phase of mitosis [133]. In rats, glutamine supplementation led to increased villus height and total intestine mass [134]. Glutamine supplementation also increased the proliferating cells in the ileum of three-week-old mice, without changing the villus height, promoting crypt cell proliferation [131]. Moreover, glutamine was shown to influence the differentiation of intestinal stem cells, as well as of Paneth cells [135]. Counts of enteroendocrine cells and goblet cells were increased, indicating enhanced differentiation [136,137].

The glycosaminoglycan hyaluronic acid is an integral part of the extracellular matrix, synovial fluids, and supports tissue hydration [138,139]. In inflammatory states, the amount of hyaluronic acid is increased [140,141]. Therefore, Riehl and coworkers investigated whether hyaluronic acid is involved in the regulation of epithelial proliferation and mucosal growth. They found out that a blocking peptide, preventing binding of hyaluronic acid to its receptors, decreased both, small intestine and colon length. Conversely, hyaluronic acid supplementation increased villus height and crypt depth in the small intestine, stimulated epithelial proliferation, and increased the number of epithelial cells. Exogenous hyaluronic acid also favored differentiation into the absorptive cell lineage, whereas goblet cells, enteroendocrine cells, and Paneth cells were reduced [142].

Not only the presence of dietary components, but also their absence has a great impact on gut morphology. Calorie restricted mice showed shorter villi and decreased numbers of enterocytes but did not increase apoptotic cell frequency [143]. Strikingly, the intestinal stem cell counts, as well as the Paneth cell counts, were significantly increased, indicating that caloric restriction enhances self-renewal of ISCs, supported by Paneth cells [143,144]. Taken together, these studies show the importance of diet composition, dietary factors, and caloric intake for balanced intestinal tissue homeostasis.

#### 4.1.2. Impact of Innate Immune Functions

Intestinal epithelial cells express pattern-recognition receptors (PRR), which are highly conserved molecules that detect pathogen-associated molecular patterns (PAMPs), including lipopolysaccharides (LPS), flagellin, and peptidoglycans [30,145] Two important families of PRR are Toll-like receptors (TLRs) and nucleotide-binding oligomerization domain-like receptors (NLRs) [146]. Intestinal TLR signaling is important in the regulation of gut homeostasis, influencing epithelial cell proliferation and differentiation, tight junctions, release of antimicrobial peptides, and induction of pro- or anti-inflammatory responses [82,147]. TLR signaling was detected in enterocytes, Paneth cells, and goblet cells, as well as in enteroendocrine cells [147,148]. To give an example, TLR4 activation in enterocytes results in the prevention of enterocyte migration into an intestinal wound, in a Ras homolog family member A (RhoA) and phosphoinositide-3-kinase dependent manner [149]. The production of mucus is upregulated in goblet cells upon TLR activation [150]. TLR4 is also expressed in intestinal stem cells and its activation impacts the regulation of proliferation capacities. TLR4 activation suppresses Wnt signaling [151] and increases epithelial differentiation in goblet cells by inhibiting Notch-signaling [152,153]. Several studies demonstrated that interactions between commensal bacteria and TLRs expressed in the intestine are crucial for epithelial homeostasis, controlling epithelial proliferation, and survival as well as barrier maintenance [154,155].

Not only are ISCs and Paneth cells present in the crypt-villus unit, but also connective tissue cells, lymphocytes, macrophages, and neurons, regulating homeostasis of stem cell self-renewal and differentiation via paracrine signals [156]. Immune cells further contribute to intestinal epithelial repair upon injury, for example in an interleukin (IL)-22-dependent manner. In small intestine enteroids, which were cocultured with IL-22 or IL-22 producing innate lymphoid cells, Lgr5+ ISCs were expanded and organoid size was increased [157]. Neither the Wnt/beta-catenin nor the Notch pathway were upregulated in IL-22 co-cultured organoids, indicating a Paneth and stromal cell-independent pathway. IL-22 induced signal transducer and activator of transcription-3 (STAT-3) phosphorylation, implying an essential role of STAT-3 signalling in IL-22 dependent epithelial regeneration [157]. Upon intestinal inflammation, IL-22 is able to enhance MUC1 levels and recovery of goblet cells [158]. Other Th2-induced cytokines, such as IL-4, -6, -9, -10, and -13, also regulate mucin production, wheras IL-4 and -13 in worm infections increase goblet cell proliferation by activation of STAT-6 signaling [159,160,161,162]. Furthermore, IL-6 secreted by intraepithelial lymphocytes (IELs) promotes intestinal epithelial proliferation [163]. On the other hand, proinflammatory cytokines including tumor necrosis factor-alpha (TNF-α) and interferon-gamma (IFN-γ) suppress β-catenin dependent epithelial cell proliferation [164].

Colony-stimulating factor-1 (CSF1), a factor secreted by macrophages, was shown to support Paneth cell maintenance [165]. In addition, macrophages are important sources of Wnt signals, ensuring ISCs survival, epithelial repair, and mouse survival upon radiation injury [166]. Macrophages, which are more abundant in the colon than in the small intestine, reside in close association with intestinal epithelial cells [167,168]. Depletion of macrophages resulted in massive apoptosis of epithelial cells in the distal colon, disrupting barrier function [169]. Moreover, IELs play an important role in the maintenance of gut homeostasis. A certain subset of IELs was shown to produce keratinocyte growth factor, aiding intestinal repair [170]. These studies show the important role of innate immune receptors and downstream signaling, as well as innate immune cells in intestinal epithelial repair and the maintenance of gut homeostasis.

#### 4.1.3. Impact of the Gut Microbiome

The gut microbiome can affect the intestinal cells via metabolites produced by gut bacteria (e.g., SCFAs via fermentation of dietary fiber) and through the recognition of microbial patterns in intestinal epithelial cells and related downstream signaling [171]. The gut microbiome can be viewed as an organ, equipped with a myriad of metabolic functions, producing various metabolites with numerous roles for the host [172]. The compositon of this metabolically active organ is largely modified by dietary changes. However, at present, it is unknown if long-term dietary interventions result in permanent alterations of the gut microbiota. Therefore, it is important for the design of nutrition studies to compare long-term dietary interventions and to plan long-term follow-ups of short-term dietary interventions [173]. Evidently, microbial metabolites also influence host metabolism [174]. SCFAs are abundant metabolites that affect intestinal homeostasis [175]. SCFAs are fatty acids with fewer than six carbon atoms, produced by bacterial fermentation of dietary fiber. The most abundant members are acetate, butyrate, and propionate [176]. Propionate contributes to gluconeogenesis in the liver and satiety signaling [177]. Butyrate is the main energy source for colonocytes, can induce apoptosis in colon cancer cells, maintains the oxygen balance in the gut thereby preventing microbial dysbiosis, activates intestinal gluconeogenesis, and is important for glucose and energy homeostasis [177,178,179]. Acetate, which is the most abundant of the three mentioned SCFAs, plays a key function in lipogenesis and is involved in cholesterol metabolism [180,181]. SCFAs are also involved in regulation of colonic epithelial cell growth and differentiation [182]. Depletion of microbiota by antibiotics results in a significant decrease of propionate and butyrate, accompanied by a downregulation of genes involved in fatty acid metabolism, indicating that enterocytes are not using these as an energy source. Instead, the enterocytes use ketone bodies and anaerobic glycolysis to generate energy [183].

Two pathways for SCFA signaling have been described: through G protein-coupled recpetors (GPCRs) GPCR43 (also known as free fatty acid receptor 2, FFAR2), GPCR41 (also known as free fatty acid receptor 3, FFAR3) and GPR109A (also known as hydroxycarboxylic acid receptor 2, HCA2) [174]. It is currently unknown which downstream pathways are preferentially activated. For example, GPCR41 and GPCR43 can initiate a proinflammatory program through MAPK-activation, but GPCR43 also is able to engage in an alternative, anti-inflammatory pathway through inhibition of NF-κB activity [184,185]. SCFAs are also able to induce NLRP3 inflammasome signaling in intestinal epithelial cells, activating important cell survival and repair mechanisms through GPCR43 and GPCR109A [186]. Moreover, SCFAs increase differentiation to goblet cells and upregulate their mucus production [187,188].

Of note, butyrate is also directly recognized by nuclear protein peroxisome proliferator-activated receptor gamma (PPAR-y) in colonocytes [189]. Binding of butyrate to that receptor results in a shift to fatty acid oxidation and oxidative phosphorylation in colon epithelial cells, resulting in high oxygen consumption, epithelial hypoxia, and anaerobiosis in the lumen, preventing the growth of facultative anaerobic bacteria [179,190,191]. Lee and coworkers demonstrated a crucial role for lactic acid-producing symbiotic bacteria, such as Bifidobacterium and Lactobacillus spp. Feeding of LAB probiotics to mice resulted in deeper crypts, increased number of Lgr5+ ISCs, Paneth cells, goblet cells, and Ki67-positive cells in the small intestine. Symbiont-derived lactate stimulates Wnt/β-catenin signaling of Paneth cells via the Gpr81-receptor and intestinal stromal cells, resulting in ISC proliferation [192]. Interestingly, the probiotic bacterium *Lactobacillus rhamnosus GG* supports cell renewal and mucosal repair upon dextran sodium sulfate-induced colitis via reactive oxygen species (ROS) production in epithelial cells [193].

The presence or absence of gut microbiota has a major impact on host gut morphology but seems to be region-specific. In germ-free mice, the following changes in gut morphology are reported: decreased total mass [194,195,196], increased intestinal length [97], longer duodenal villus structures [197], longer jejunal villus structures [96], shorter ileal villi [198], shorter crypts in the entire small intestine [198,199], and an enlarged cecum with thinner cecal walls and shorter villi [200,201,202,203] as compared to their conventionally-raised counterparts. In addition, the mucosal layer is thinner and less stable in germ-free mice [204,205,206], accompanied with lower numbers of goblet cells [207]. Exposure to bacterial components, such as lipopolysaccharides (LPS) or peptidoglycans, can suffice to establish conventional mucus characteristics [208].

Differences between germ-free and conventionally raised mice are independent of epithelial proliferation, intestinal stem cell number, and cycling status in the jejunum, as shown by Schönborn and coworkers [96]. Preidis and coworkers gavaged *Lactobacillus reuteri*, a human-derived probiotic bacterium, to neonatal specific-pathogen-free mice. They found an increase in enterocyte migration, proliferation and crypt depth, in all three parts of the small intestine, paired with unchanged villus length. Sequencing the gut microbiota showed that *Lactobacillus reuteri* gavage leads to an enhanced phylogenetic diversity in the gut microbiome of neonatal mice [209]. Collectively, these studies underline the direct and indirect roles of commensal microbiota in the regulation of intestinal tissue homeostasis.

### 4.2. Effects on the Intestinal Microvasculature

In the small intestinal villus structures, the integrity of intricate capillary networks and lacteals of the lymphatic system is crucial for efficient tissue oxygenation, nutrient uptake, and transport [210,211,212]. Upon inflammatory conditions such as in inflammatory bowel disease, the intestinal microvasculature is severely perturbed [213,214,215]. Interestingly, mouse studies demonstrated that high-fat diet conditions promote tumor angiogenesis in colon cancer [216]. Furthermore, the gut-resident microbiota constitute a rich source of diverse inflammatory stimuli, interfering with nutrition and host metabolism, thus affecting postnatal development of capillary networks [217]. As the intestinal vasculature serves nutrition and is therefore an actuating variable of many intestinal and metabolic functions [218], it is essential to gain insights into how the interplay between nutrition, microbiome, and innate immune functions acts on the adaptation of mucosal capillaries.

#### 4.2.1. Impact of Nutrition

While the impact of different diets on intestinal immune cell phenotypes was extensively characterized during the past decade [219,220], surprisingly little is known on the role of defined diets on gut vascular phenotypes. Diet has a clear impact on intestinal microvascular inflammation. It was demonstrated that high-fat diet (HFD) feeding enhances the adherence of T-lymphocytes in the small intestinal microvessels [221]. Furthermore, HFD promotes disruption of the gut vascular barrier [222]. After antibiotic treatment, Western diet-induced vascular dysfunction can be reversed, demonstrating an important link between diet, microbiota, and the circulatory system [223]. Upon inflammatory conditions of 1,4,6-trinitrobenzene sulfonic acid-induced colitis, n3-polyunsaturated fatty acids reduced endothelial vascular cell adhesion molecule-1 and vascular endothelial growth factor receptor-2 staining in the rat colon [224,225]. Interestingly, small bowel resection evokes intestinal adaptation, comprising the lengthening of villus structures, crypt deepening, and an increase in the density of villus capillaries [218,226]. In contrast, the mucosal lymphatic area is reduced following small bowel resection [227]. In conclusion, Western diet is a strong factor initating or exacerbating vascular dysfunction but still molecular mechanism of nutrition-dependent vascular adaptation in the gastrointestinal tract awaits further investigation.

#### 4.2.2. Impact of Innate Immune Functions

Human microvascular endothelial cells express pattern recognition receptors, such as Toll-like receptors (TLRs) and nucleotide-binding and oligomerization domain (NOD)-like receptors, which were shown to promote angiogenesis in cell culture experiments [228,229,230]. Of note, intestinal endothelial cells have the capacity to develop tolerance against bacterial endotoxins [231]. Stimulation of human intestinal microvascular endothelial cells (HIMEC) by bacterial ligands resulted in the activation of TLR2/6, TLR4, NOD1, and NOD2. Subsequent signaling cascades involving mitogen-activated protein kinases (MAPK) and nuclear factor kappa-B (NF-κB) pathways, as well as phosphorylation of the focal adhesion kinase (FAK), and, most notably, upregulation of vascular endothelial growth factor receptor 2 (VEGFR2) mediates a pro-angiogenic response [230]. However, it was observed that selective inhibition of TLR2 in human umbilical vein endothelial cells (HUVECs) via antibody therapy also induced angiogenesis [232]. The highly conserved TLR4 signals through the TLR-adaptors myeloid differentiation primary response protein 88 (MyD88) or TIR-domain-containing adapter-inducing interferon-β (TRIF) pathways. *In vivo*, mice double-deficient for the TLR-adaptors MyD88 and TRIF displayed markedly reduced villus vascularization, indicating a role for these pattern recognition receptors in the development of intestinal capillaries [233]. In fact, TLRs are critically involved in the immune response of endothelial cells but also in the process of tissue repair by responding to damage signals and regulating the intricately branched vasculature [234]. Furthermore, chemokines, as part of the innate immune response, influence mucosal angiogenesis in the intestine. Interestingly, mice deficient in CXC-ligand (CXCL)-5 showed an impaired adaptation of submucosal capillary density following small bowel resection, indicating a role for the attraction of innate immune cells in the vascular adaptation of intestinal capillaries [218]. Another study demonstrated that chemotaxis, proliferation, and tube formation of cultured human microvascular intestinal endothelial cells are mediated via CXCL-12 signaling, a chemokine that is prominently expressed in human colonic mucosal microvessels [235]. Besides pattern recognition receptors (PRRs), innate immune cells participate in microvasculature remodeling. For instance, self-maintaining gut macrophages colonize distinct gut niches closely localized to blood vessels. Depletion of gut macrophages demonstrated morphological deficits in the submucosal vasculature, thereby leading to vascular leakage [236].

The impact of innate immune signaling pathways on mucosal capillaries is also relevant in intestinal disease phenotypes. For example, in Crohn’s disease, therapeutic silencing of TNF-α signaling reduced the immunohistochemical expression of the vascular marker cluster of differentiation 31 (CD31; PECAM-1) [237]. Moreover, it was suggested that the neuropeptide substance P, via the induction of the cysteine-rich angiogenic inducer-61 in colonic epithelial cells, can promote mucosal angiogenesis in colitis [238]. Interestingly, in colorectal cancer, tube formation assays as well as immunohistochemistry analyses on an orthotopic xenograft nude mouse colorectal cancer model demonstrated that lymphangiogenesis and lymph node metastasis was promoted by lipopolysaccharide via vascular endothelial growth factor-C (VEGF-C) signaling [239].

**Figure 4:**
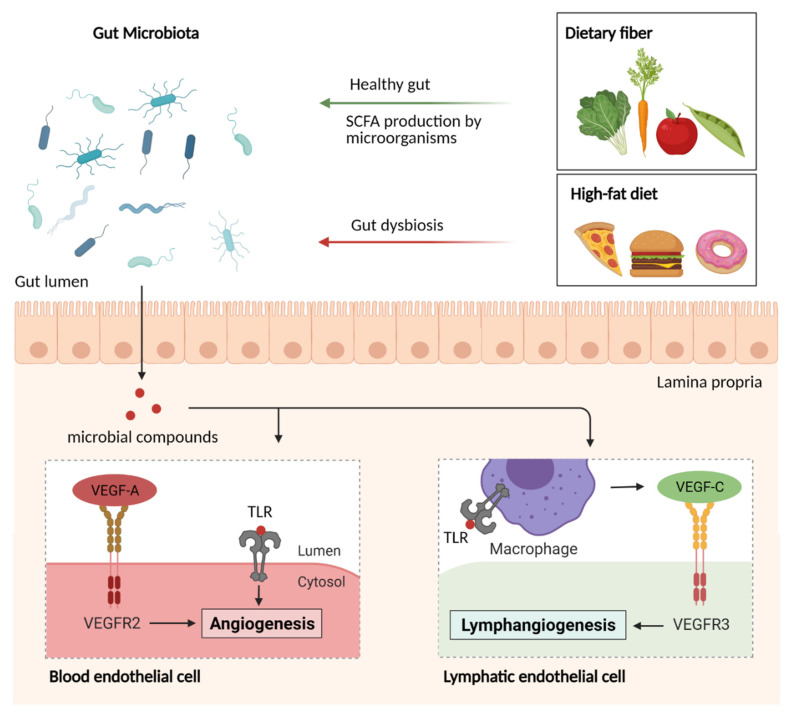
Impact of diet, microbiota, and innate immunity on intestinal endothelial cells. Nutrient uptake shapes the gut microbiota composition. Microbial compounds induce angiogenesis or lymphangiogenesis by evoking a Toll-like receptor (TLR)-mediated immune response. Angiogenesis is induced via vascular endothelial growth factor (VEGF)-2 and -3 signaling. Created with *BioRender* [63].

Collectively, PRR, specific immune cells as well as chemokines play a crucial role in the growth and maintenance of the intestinal vasculature. Activation of TLRs on endothelial cells via microbial compounds induce VEGF-A and VEGF-C production and are thus involved in the endothelial and lymphatic endothelial cell development, respectively (Figure 4). Disturbance of the innate immune function clearly evokes vascular aberrations and even vascular diseases.

#### 4.2.3. Impact of the Gut Microbiome

The gut microbiota is a pivotal modifier of intestinal vascular host physiology [217,218]. This densely colonized microbial ecosystem does not only affect innate immune signaling and mucosal immune phenotypes but also represents a rich source of metabolites that are taken up by the host [30,240,241]. The absence of gut commensals in germ-free mice is accompanied by an underdeveloped villus capillary network [31]. Remarkably, 10 days of colonization with a cecal gut microbiota from a conventionally raised mouse or the gut resident bacterium *Bacteroides thetaiotaomicron* in germ-free mice are sufficient to increase the complexity of the villus capillaries to the extent observed in conventionally raised mice. Colonization with a gut microbiota resulted in the activation of protease-activated receptor-1 (PAR1) via a tissue factor-dependent mechanism, promoting the formation and stabilisation of intricate capillary networks by angiopoietin-1 signaling [32]. In addition to epithelial signaling cues, intestinal subepithelial myofibroblasts were demonstrated to possess angiogenic properties in a gut-on-a-chip model [242]. Besides blood capillaries, lymphatic vessels in the small intestine have a pivotal role in transporting dietary lipids as well as immune cells [243]. Germ-depletion with antibiotic cocktails and recolonization in mice unveiled that macrophages in the lamina propria are a key factor for the maintenance of lacteal integrity. Upon TLR-MyD88-dependent recognition of microbes, those villus macrophages secrete VEGF-C, therefore contributing to the maturation of the gut mucosal lymphatic system [33].

Inflammation of the intestine results in the loss of microbial compounds through the epithelium and activation of endothelial cells [244,245,246]. In particular, aging is a high-risk factor for chronic systemic inflammation referred to as inflammaging. In aging mice, the proportion of M2-like macrophages, which secrete TNF-α in the lower gut, increases progressively. Hence, the upregulation of angiopoietin-2, the counterpart of the microvessel stabilizing angiopoietin-1, induced deterioration of microvascular structures [247,248]. In inflammatory bowel disease (IBD), an immune-driven microvascular remodeling occurs under the influence of VEGF-A [249]. Newly emerging data have revealed that expression patterns of PRR, namely TLR4, are not regulated upon contact with microbial compounds as expected, but independently determined prior to birth. This segment-specific organisation of PRR coincides with different pathological phenotypes in subsets of IBD [250]. During neoplastic transformation, tumor cells switch to an angiogenic phenotype, accompanied by an imbalance of positive and negative regulators [251]. To sustain tumor growth, different subsets of immune cells such as macrophages, granulocytes, mast cells, and natural killer (NK) cells regulate the formation and remodelling of blood vessels by releasing angiogenic factors [252,253]. Hereby, in gastrointestinal tumors, infiltrates of neutrophil granulocytes have been observed to produce VEGF [254,255], CXCL8 [256], CXCL1 [257], and matrix metalloproteinase-9 (MMP-9) [258]. Furthermore, dysbiosis induced by high-fat diet leads to the disruption of the gut-vascular barrier and to bacterial translocation to the liver, promoting the development of non-alcoholic steatohepatitis (NASH) [222].

Alltogether, these studies highlight several findings in the interaction of the gut microbiota with the intestinal vasculature via TLRs, PAR1, or various immune cell subtypes such as macrophages. Here, a particularly valuable tool are germ-free mice. Elucidating the precise mechanisms behind nutrition, innate immune signalling and microbiota should provide new therapeutic approaches against vascular inflammatory diseases.

### 4.3. Effects on the Enteric Nervous System

The gut microbiota is known to regulate thousands of genes, some of which affect gut morphology [259]. Commensal microbiota is critical for maintenance of the intestinal barrier, which in turn protects the mucosal tissues from toxins and proinflammatory molecules. Nonetheless, there are several ways in which intestinal microbes influence the host through neurochemicals, microbial metabolites as well as through biosynthesis of multiple small molecules and cellular components [260]. Given the relatively close proximity of gut microbiota to the ENS, a direct impact of microbial commensals on the neuronal tissue has to be considered [261].

#### 4.3.1. Impact of Nutrition

**Figure 5:**
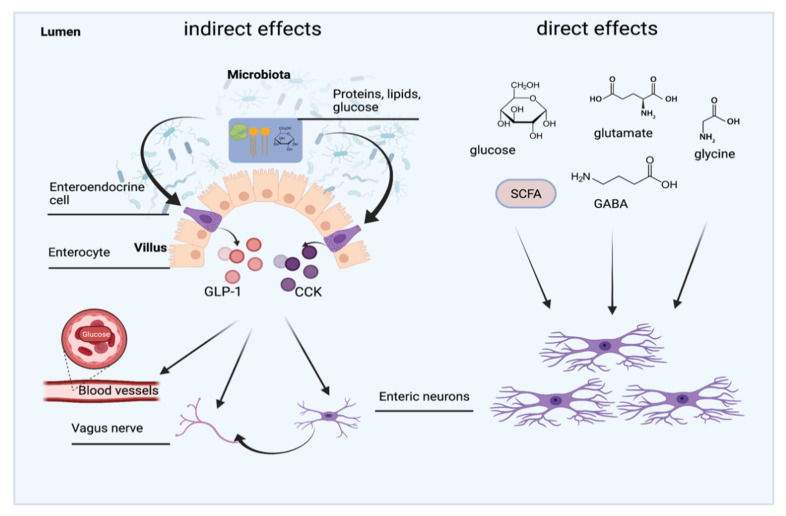
Indirect and direct effects of microbiota and nutrition on the ENS. Created with *BioRender* [63].

While development of the ENS takes place mostly in early life, achieved disorders or physiological parameters can affect the ENS throughout the whole life span. For instance, guinea pigs were used as a model to find out how hunger and satiety can regulate peristalsis and general enteric nerve activity [262]. The experiments showed that peristaltic activity was increased in ileal segments of animals that were re-fed after an overnight fasting. Myenteric neurons of the re-fed guinea pigs were also hyperresponsive to high K^+^- induced depolarisation, anorexigenic molecules such as cholecystokinin-8 (CCK-8), and simultaneously less responsive to ghrelin. Therefore, it was assumed that propulsive activity was regulated by the ENS [263]. Cholecystokinin (CCK) is a peptide hormone synthetized by enteroendocrine I-cells and by the enteric and central nervous system [264]. CCK-1 and -2 receptors are also expressed in enteric neurons and have been shown to be involved in nutrient-induced segmentation (nutrient mixing) in the guinea pig’s gut [265]. Ghrelin, another gastrointestinal hormone, which is expressed in gastric endocrine cells, has been shown to stimulate the gastrointestinal motility in rat and human gastrointestinal tract [266]. Moreover, it was reported that preabsorptive nutrients induce the release of glucagon-like peptide-1 (GLP-1) and CCK through activation of enteroendocrine cells. GLP-1 and CCK, in turn, interact with the vagal nerve and enteric neurons and consequently regulate glucose and energy homeostasis (Figure 5) [267,268]. Direct effects of nutrients on the ENS have also been reported in previous studies. Enteric neurons can directly interact with nutrients via nutrient sensing by enteroendocrine cells (Figure 5). Several transporters and receptors are expressed by enteric neurons and enable the process of nutrient sensing [269]. For instance, this includes the sodium glucose co-transporter-1 (SGLT-1), the Na^+^-D-glucose transporter, which is responsible for glucose sensing, G-protein coupled receptor (GPR) 41 for SCFA sensing, dipeptide transporter Pept2 as well as amino acid receptors, which are activated by glutamate, glycine, or GABA for protein, peptide sensing [265].

In summary, these studies revealed several mechanisms of nutrient communication with the ENS. However, the input of the ENS on nutrient-stimulated changes of the gut still remains a subject of future investigation.

#### 4.3.2. Impact of Innate Immune Functions

It has been shown that small molecules from gut microbiota in humans are involved in numerous biological activities and can affect the physiology of the host [270]. It has been well investigated that TLRs play an important role in the development of the central nervous system (CNS) [271]. The intestinal microbiota can also modulate ENS functions through Toll-like-receptors (TLRs). TLR2, for example, is expressed in intestinal smooth muscle layers, enteric neurons, and glial cells. Interestingly, it has been shown to play a crucial role in ENS homeostasis [272]. TLR2-deficient mice showed abnormalities in the ENS structure, intestinal dysmotility as well as reduced levels of glial cell line-derived neurotrophic factor (GDNF) [272]. Moreover, bacterial products such as lipopolysaccharides (LPS) can influence the ENS, as the LPS-induced activation of TLR4 increases neural survival and gastrointestinal motility in mice [34].

Although most immune cells are concentrated in the lamina propria, the main ENS networks, the myenteric and submucosal plexuses, harbor a large population of muscularis macrophages and mast cells [273,274]. Gastrointestinal macrophages have been shown to control the gastrointestinal motility through interaction with enteric neurons [275]. Muscularis macrophages secrete the growth factor bone morphogenic protein 2 (BMP2) to support the enteric neurons. The enteric neurons, in turn, produce the specific growth factor CSF1, which promotes the muscularis macrophage homeostasis [275]. In particular, while the enteric nervous system seems dispensable for muscularis macrophage colonization during development [276], β2-adrenergic receptor-positive muscularis macrophages reside closely to active, firing enteric neurons postnatally [277]. Enteric infection elicited changes in gene expression of muscularis macrophages via neuron-derived adrenergic signaling, resulting in a more tissue-protective phenotype [278]. Other gut-resident innate immune cells, such as mast cells, are located in mucosal and submucosal tissues throughout the gastrointestinal tract and play a key role in the inflammatory process. Their proximity to the enteric nerves enables bidirectional communication [279]. This crosstalk takes place when neurons secrete neuropeptides such as calcitonin-gene related peptide (CGRP), substance P, vasoactive intestinal protein (VIP), and corticotropin-releasing hormones (CRHs), which initiate mast cell degranulation and activate them [279]. The mast cells then provide neuronal homeostasis through serotonin, tryptase, and histamine production [279,280]. Former studies suggest that the positive feedback-loop, created through a bidirectional communication, could cause neurogenic inflammation. Furthermore, the irritable bowel syndrome (IBS), as well as inflammatory bowel disease (IBD), and inflammations caused through food allergy are associated with direct mast cell activation by bacterial antigens [279,281].

#### 4.3.3. Impact of the Gut Microbiome

While the majority of the ENS components develop during embryogenesis, the colonization with microbiota and maturation of enteric immune structures occur during the phase of postnatal neurogenesis [282]. Experiments on germ-free mice showed that the lack of intestinal bacteria in early life induces structural changes in the myenteric plexus in the germ-free jejunum and ileum [283]. The study by Collins et al. demonstrates a decrease in nerve density and number of neurons per ganglion, as well as reduced gastrointestinal motility in jejunum and ileum of germ-free mice. These structural defects have not been observed to be lethal but are responsible for the atypical gut motility [283]. De Vadder et al. demonstrated a crosstalk between gut microbiota and ENS via activation of the 5-HT_4_ receptor [284]. Through colonization of germ-free mice with normal microbiota from conventionally raised mice as well as the release of serotonin (5-HT), the ENS structure was modified, and propulsive activity increased. Moreover, the germ-free animals showed reduced innervation in the colonic epithelium, which was restored to normal levels 15 days after colonization [284,285]. Interestingly, the impact of many individual gut microbes on gastrointestinal transit has also been reported in recent years [286,287,288]. Such observations are commonly based on microbial manipulations in germ-free animals, which enable the analysis of the influence of specific microbes on the intestine through enteric intrinsic primary afferent neurons. *Bacteroides thetaiotaomicron*, for instance, was reported to increase mucosal innervation in the colon *of B. thetaiotaomicron* monocolonized mice in comparison to germ-free mice and regulate colonic propulsive activity [288]. Taken together, these studies demonstrate the significance of healthy gut microbiota in the development and function of the ENS. Understanding of molecular mechanisms underlying this bidirectional communication may provide new therapeutic targets for gastrointestinal inflammatory disorders.

### 4.4. Effects on Intestinal Smooth Muscle Cell Layers

An important function of the gut is the transport of food for optimal exposure to digestive enzymes [289]. The contractility of the intestine is regulated by the smooth muscle, which can develop slow but long contractions, mixing and propelling the intraluminal contents, thus enabling digestion. The morphology of the gut wall is similar in most vertebrates, comprised of two smooth muscle layers, an outer thin layer of cells forming the longitudinal smooth muscle layer, and a thicker perpendicular layer inside the longitudinal muscle, the circular smooth muscle layer [289].

#### 4.4.1. Impact of Nutrition

Intestinal smooth muscle cells play a critical role in the remodeling of the intestinal structure and the functional adaptation after bowel resection. One of the first studies pinpointing the differential effect of diet on the intestinal smooth muscle of rats showed that the circular muscle cell size increased (22.5% in the proximal and 77.9% in the distal colon) after consumption of wheat bran for nine weeks while a four-week consumption of either oat bran, pectin, or guar resulted in a 20.6% decrease in the proximal jejunum for the first two fibers and a 43% decrease in the proximal colon after pectin consumption [290]. This work indicated that the effects of high-fiber diet on the intestinal muscle size depend on the type of fiber consumed. Moreover, SCFAs, the end product of dietary fiber fermentation in the colon and especially butyrate, modulate not only the proliferation of intestinal smooth muscle cells but also the expression of collagenous and cytoskeletal protein in primary intestinal smooth muscle cell culture and in vivo in piglets [291,292]. On the other hand, dietary fiber deficiency impairs the muscle contractile response in rat distal colon in a neuronal-dependent manner [293].

The effect of diet on smooth muscle cells has also been addressed in the context of parenteral nutrition. A study using rats with parenteral nutrition demonstrated that L-glutamine is the preferred fuel for the jejunal smooth muscle, whereas, in the absence of other amino acids, exogenous glutamine prevents the atrophy of the gut musculature in rats [294]. Zhu et al. studied the effect of choline in rats with parenteral nutrition and found that choline supplementation protects the smooth muscle cells from injury, diminishing the progression of duodenal motor disorder [295]. Vrabcova et al. investigated the effect of liquid nutrition in the morphology of the gut, proving that the muscle layer thickness is independent of the form of the food intake [296].

#### 4.4.2. Impact of Innate Immune Functions

Unspecific innate immunity of the gut is the first line of defense that effectively prevents infections caused by invading pathogens. Elements of the gut innate immunity include the mucosal barrier, secretory molecules, and cellular components. Gut-resident muscularis macrophages, which serve as central gut immune cells, are crucial for maintaining gastrointestinal homeostasis at steady-state and are important for protection from certain pathogens [297,298]. Muscularis macrophages express TLRs, which are stimulated by pathogen-associated molecular patterns (PAMPs) and damage-associated molecular patterns (DAMPs). Upon TLR-stimulation, the gut macrophages are involved in enhanced production of various inflammatory cytokines such as IL-6 and TNF-α, in addition to promoting naive T-helper cells for IFN-γ production [299]. Recent studies revealed that these inflammatory cytokines have an essential role in inducing and modulating platelet-derived growth factor (PDGF-BB), which is identified to be expressed in the epithelium and serves as a key mitogen for intestinal smooth muscle cells [298,300]. Experiments on the rat jejunum using *Trichinella spiralis*-induced inflammation also demonstrated that an inflamed intestine leads to the thickening of the smooth muscle layers in this segment compared to the non-inflamed ileum. This showed that the thickening of smooth muscle layers is caused by the hyperplasia of intestinal smooth muscle cells, which phenotypically differ from other smooth muscle cells. The altered phenotype of intestinal smooth muscle cells might be caused by inflammatory cytokines in the microenvironment [301]. Through their ability to establish cell-to-cell contact with intestinal smooth muscle cells, muscularis macrophages have an important role in the modulation of gastrointestinal motility [298]. Consequently, the permanent alteration of intestinal smooth muscle cell phenotype in chronic inflammation may lead to intestinal dysmotility, while the growth of intestinal smooth muscle cells serves as an important part of structure formation in human inflammatory bowel disease.

#### 4.4.3. Impact of the Gut Microbiome

The gut microbiota influences gastrointestinal motility and specifically the smooth muscle cell layer in various ways [302]. Apart from the indirect effect of the microbiota-derived inflammatory mediators, stimulating the production of smooth muscle cell-derived neurotrophic factors [303], the microbiota also directly influences smooth muscle cell function via metabolic products. For instance, SCFAs, the bacterial fermentation products of dietary fibers stimulate ileal motility in humans [304]. Butyrate-producing bacteria are involved in gut motility, with low concentration to stimulate and high concentration to have an inhibitory role [305]. A study including patients with chronic constipation found that, while both, the circular and the longitudinal muscles of the patients, exhibited increased contraction amplitudes as compared to the control group, the microbiota composition of the patients showed a low abundance of butyrate-producing genera, i.e., Roseburia, Coprococcus, and Faecalibacterium [306].

In summary, the influence of smooth muscle cell layers on the gut morphogenesis is diverse and influenced by many external and internal factors. Smooth muscle cell layers on the one hand contribute to fundamental functions of intestinal tissue homeostasis and cell survival. On the other hand, increase of various inflammatory cytokines during infection can lead to hyperplasia of the smooth muscle cell layers, promoting the development of inflammatory bowel diseases.

## 5. Challenges and Limitations

Although the development of the alimentary tract in mice has been well-defined by excellent developmental studies and a wealth of transgenic mouse models, our current knowledge on the effects of diet, microbiota, and immune pathways, interfering with morphogenetic signaling of the intestine, remains sparse. Mice and men are not fully comparable by means of intestinal architecture and their gut resident microbiome. However, mice certainly are a suitable model to understand adaptive processes in intestinal tissue homeostasis. Hence, further nutritional studies, i.e., with chemically defined diets but also experimentation with gnotobiotic mouse models are needed to resolve this complex interplay. At present, a clear limitation is the lack of studies on human gut morphology across scales, considering factors such as genetics, nutritional habits, or age. Of note, pre- and probiotics emerge as modulators of the gut microbiota, affecting many functional aspects in the intestinal mucosa, but this topic is beyond the scope of this review [307]. Furthermore, fermentable oligosaccharides and polyols are important nutrients that influence intestinal phenotypes by shaping the composition of the gut microbial ecosystem. However, as this topic was recently discussed by other excellent review articles [308,309], we did not deal with these nutrients. In addition, our review does not focus on the intestinal endocannabinoid system, which is involved in the regulation of gut barrier function and the leakage of microbiota-derived lipopolysaccharides, a regulating element in adipose tissue plasticity [310].

## 6. Conclusions

Although at birth the intestine is fully developed and the intestinal mucosa comprises a robust epithelial barrier that is closely located to an intact capillary network that interferes with the ENS, this organ warrants a high degree of flexibility and adapts to various environmental stressors, such as nutrition and the colonization with a gut microbiota. Steady-state turnover of the epithelium, potentially driven by mitotic pressure from the crypts, was observed in many organisms. Insects such as *Lepidoptera* show not only increased differentiation and proliferation of stem cells during molting but also in consequence to pathogenic episodes. While rapid epithelial renewal and the morphologic adaptation of the intestinal mucosa are instrumental to keep commensals at bay, we are just beginning to appreciate how this mutualistic relationship between microbiota and tissue morphogenesis integrates into host physiology. Considering its vital role in nutrient harvest, the intestine is an organ where “form follows function”. Importantly, the respective reactions are not solely driven by the endogenous immune system of the host, dependent on nutrition, but they are tightly controlled by gut commensals. The continuous self-renewal of the gastrointestinal tract is therefore based on a multi-parametric, dynamic, and adaptive process, not only driven by various cell-types but also multi-organismic.

To fully elucidate these processes, a reductionist approach, based on gnotobiotic mouse models combined with bioinformatic modeling approaches, will be needed. One attempt to rather eliminate one dimension of complexity is the analysis of germ-free or monocolonized mouse models. The gut microbiota is a superorganism that influences host physiology even in remote organs. Whereas the interplay of gut commensals with innate immune cells and pattern recognition receptors are increasingly recognized as an actuating variable on epithelial renewal, cell differentiation and gut morphology, it remains unexplored whether the gut microbiota can affect morphologic adaptation processes via additional signaling pathways. So far, the communication between the epithelial lining of the intestine with the endothelial cells of villus capillaries or the submucosal and myenteric plexus of the ENS is poorly resolved. Furthermore, the investigation of the age-dependency of adaptive gut morphogenesis and how this is linked to fuctional traits is an issue of growing interest.

## Data Availability

Not applicable.
